# Direct and indirect costs and cost-driving factors in adults with tuberous sclerosis complex: a multicenter cohort study and a review of the literature

**DOI:** 10.1186/s13023-021-01838-w

**Published:** 2021-06-02

**Authors:** Johann Philipp Zöllner, Janina Grau, Felix Rosenow, Matthias Sauter, Markus Knuf, Gerhard Kurlemann, Thomas Mayer, Christoph Hertzberg, Astrid Bertsche, Ilka Immisch, Karl Martin Klein, Susanne Knake, Klaus Marquard, Sascha Meyer, Anna H. Noda, Felix von Podewils, Hannah Schäfer, Charlotte Thiels, Laurent M. Willems, Bianca Zukunft, Susanne Schubert-Bast, Adam Strzelczyk

**Affiliations:** 1 Epilepsy Center Frankfurt Rhine-Main, Department of Neurology, Goethe-University Frankfurt, Schleusenweg 2-16, 60528 Frankfurt am Main, Germany; 2 Center for Personalized Translational Epilepsy Research (CePTER), Goethe-University Frankfurt, Frankfurt am Main, Germany; 3 Klinikum Kempten, Klinikverbund Allgäu, Kempten/Allgäu, Germany; 4 Department of Pediatrics, Helios Dr. Horst Schmidt Clinic Wiesbaden, Wiesbaden, Germany; 5 Department of Pediatrics, University Medicine Mainz, Mainz, Germany; 6 St. Bonifatius Hospital, Lingen, Germany; 7 Epilepsy Center Kleinwachau, Radeberg, Germany; 8 Department of Neuropediatrics, Vivantes Klinikum Neukölln, Berlin, Germany; 9 Department of Neuropediatrics, University Hospital for Children and Adolescents, Rostock, Germany; 10 Epilepsy Center Hessen and Department of Neurology, Philipps-University Marburg, Marburg (Lahn), Germany; 11 Departments of Clinical Neurosciences, Medical Genetics, and Community Health Sciences, Hotchkiss Brain Institute and Alberta Children’s Hospital Research Institute, Cumming School of Medicine, University of Calgary, Calgary, AB Canada; 12 Department of Pediatric Neurology, Psychosomatics and Pain Management, Klinikum Stuttgart, Stuttgart, Germany; 13 Department of Neuropediatrics, Children’s Hospital at University Medical Center Homburg, Homburg, Germany; 14 Department of Neurology, Epilepsy Center, University Medicine Greifswald, Greifswald, Germany; 15 Division of Nephrology, Medizinische Klinik und Poliklinik IV, Klinikum der LMU München - Innenstadt, München, Germany; 16 Department of Nephrology, Klinikum Rechts Der Isar, Technische Universität München, München, Germany; 17 Department of Neuropediatrics and Socialpediatrics, University Hospital of Ruhr University Bochum, Bochum, Germany; 18 Department of Nephrology and Internal Intensive Care, Charité - University Medicine Berlin, Berlin, Germany; 19 Department of Neuropediatrics, Goethe-University Frankfurt, Frankfurt am Main, Germany

**Keywords:** TSC, Angiomyolipoma, Seizure, Epilepsy, Subependymal giant cell astrocytoma, Costs, Sociodemographic characteristics, Genetics, Anticonvulsant, MTOR inhibitor

## Abstract

**Background:**

Tuberous sclerosis complex (TSC) is a monogenetic, multisystem disorder characterized by benign growths due to *TSC1* or *TSC2* mutations. This German multicenter study estimated the costs and related cost drivers associated with organ manifestations in adults with TSC.

**Methods:**

A validated, three-month, retrospective questionnaire assessed the sociodemographic and clinical characteristics, organ manifestations, direct, indirect, out-of-pocket (OOP), and nursing care-level costs among adult individuals with TSC throughout Germany from a societal perspective (costing year: 2019).

**Results:**

We enrolled 192 adults with TSC (mean age: 33.4 ± 12.7 years; range: 18–78 years, 51.6% [n = 99] women). Reported TSC disease manifestations included skin (94.8%) and kidney and urinary tract (74%) disorders, epilepsy (72.9%), structural brain defects (67.2%), psychiatric disorders (50.5%), heart and circulatory system disorders (50.5%), and lymphangioleiomyomatosis (11.5%). *TSC1* and *TSC2* mutations were reported in 16.7% and 25% of respondents, respectively. Mean direct health care costs totaled EUR 6452 (median EUR 1920; 95% confidence interval [CI] EUR 5533–7422) per patient over three months. Medication costs represented the major direct cost category (77% of total direct costs; mean EUR 4953), and mechanistic target of rapamycin (mTOR) inhibitors represented the largest share (68%, EUR 4358). Mean antiseizure drug (ASD) costs were only EUR 415 (6%). Inpatient costs (8%, EUR 518) and outpatient treatment costs (7%; EUR 467) were important further direct cost components. The mean care grade allowance as an approximator of informal nursing care costs was EUR 929 (median EUR 0; 95% CI EUR 780–1083) over three months. Mean indirect costs totaled EUR 3174 (median EUR 0; 95% CI EUR 2503–3840) among working-age individuals (< 67 years in Germany). Multiple regression analyses revealed mTOR inhibitor use and persistent seizures as independent cost-driving factors for total direct costs. Older age and disability were independent cost-driving factors for total indirect costs, whereas epilepsy, psychiatric disease, and disability were independent cost-driving factors for nursing care costs.

**Conclusions:**

This three-month study revealed substantial direct healthcare, indirect healthcare, and medication costs associated with TSC in Germany. This study highlights the spectrum of organ manifestations and their associated treatment needs in the German healthcare setting. *Trial registration*: DRKS, DRKS00016045. Registered 01 March 2019, http://www.drks.de/DRKS00016045.

**Supplementary Information:**

The online version contains supplementary material available at 10.1186/s13023-021-01838-w.

## Key points

- This comprehensive study measured the direct and indirect costs of individuals with TSC and their caregivers
- Mean total direct costs (healthcare and non-healthcare) were estimated at EUR 6452 over three months
- Medication, particularly mTOR inhibitors, were major direct cost components, followed by hospitalization and outpatient treatment
- Mean total indirect costs were estimated at EUR 3174 over three months; with an inability to work being the largest factor
- Total cost is driven by the number of TSC manifestations and affected organ systems

## Background

Tuberous sclerosis complex (TSC) is a rare multisystem, monogenetic disorder. The estimated incidence rate of definite or possible TSC in Germany is approximately 1:6760 to 1:13,520 live births [1]. The prevalence of TSC was generally underestimated until recently due to incomplete penetrance and the existence of considerable interindividual phenotypic variability among those affected by TSC [1–6]. In TSC, benign tumors manifest in multiple organ systems, and the clinical manifestations of TSC can vary throughout life, with tumors presenting in most organs, especially the skin, brain, and kidneys. Most individuals with TSC suffer from structural epilepsy due to the formation of cortical tubers or other cortical malformations [7]. The clinical picture for each individual may differ considerably and can range from very limited manifestations to severe impairments that require nursing assistance [4, 7]. Individuals are commonly diagnosed with TSC in response to the development of epileptic seizures, particularly the development of epileptic spasms at a young age, often within the first six months after birth [8]. Other common first findings include skin manifestations, and TSC can sometimes be suspected even before birth due to cardiac rhabdomyoma [7]. Neuropsychiatric problems, including intellectual disability, autism, sleep difficulties, and aggression are frequent in children with TSC, they have been associated with early seizure onset, epileptic spasms, and *TSC2* gene mutations, among other factors [7]. During adolescence, renal manifestations, such as angiomyolipoma (AML) and subependymal giant cell astrocytoma (SEGA) can become burdensome [9]. Renal AML tends to grow during adulthood, necessitating life-long surveillance [10]. Pulmonary manifestations such as lymphangioleiomyomatosis (LAM) almost exclusively affect adult women with TSC [11].

TSC is caused by a loss-of-function mutation in one of two tumor suppressor genes, *TSC1* and *TSC2* (ratio 1:3.4, as reported in [12]), which is inherited in an autosomal-dominant fashion. However, the majority of cases appear to occur due to de novo pathogenic variants. Genetic mosaicism and deep intronic mutations may also be causative, particularly among the 15% of cases for which definitive hereditary pathogenic variants cannot be identified, despite a definite clinical diagnosis of TSC [12]. A loss-of-function mutation in either *TSC1* or *TSC2* leads to the overactivation of the mechanistic target of rapamycin (mTOR) pathway, which results in changes in cell growth, the promotion of cell proliferation, and the disruption of cellular energy homeostasis, ultimately promoting tumorigenesis [13]. Treatment with mTOR inhibitors can address this downstream deregulation as they prevent epileptogenesis and possibly the development of other organ manifestations [14].

The burden of illness associated with TSC is considerable and can vary according to the complex and multifaceted disease manifestations [15–17]. Several studies published during the last two decades have examined the cost-of-illness (COI) and COI predictors in TSC. However, only a few have addressed both direct costs and related cost drivers, and no study has examined the indirect costs incurred by adult individuals with TSC. Furthermore, the majority of these studies evaluated individuals with TSC before the availability of mTOR inhibitors, such as everolimus, which are now used to treat various organ manifestations associated with TSC [4, 18].

Thus, the present study aimed to provide a comprehensive analysis of the direct and indirect costs and potential cost-driving factors associated with TSC by surveying a large, multicenter cohort of adults with TSC in Germany.

## Methods

### Patients and recruitment

The present study was designed as a cross-sectional, multicenter survey that enrolled individuals with TSC throughout Germany (Berlin, Bochum, Dresden [Radeberg], Frankfurt, Greifswald, Homburg, Kempten, Marburg, München, Münster [Lingen], Rostock, Stuttgart, and Wiesbaden) and through the German TSC patient advocacy group (Tuberöse Sklerose Deutschland e.V., Wiesbaden, Germany).

### Survey methods

After receiving written informed consent from the patients or their legal guardians (if applicable), all individuals with TSC were deemed eligible for study inclusion. We based the diagnostic criteria for TSC on the latest recommendations established by the 2012 international TSC consensus conference [19]. We identified seven primary manifestation categories affected by TSC, including epilepsy, structural brain defects, psychiatric, heart/circulatory system disorders, kidney and urinary tract disorders, dermatological system manifestations, respiratory system manifestations, and other manifestations [16]. The seizure and epilepsy syndrome classifications were adapted to the latest definitions established by the International League against Epilepsy (ILAE) [20, 21]. This study received ethical approval and was registered with the German Clinical Trials Register (DRKS00016045; Universal Trial Number: U1111-1229-4714). We closely followed the STROBE guidelines (Strengthening The Reporting of Observational Studies in Epidemiology) [22].

We asked individuals with TSC to complete a retrospective questionnaire based on their experiences during the previous three months. The questionnaire was validated in earlier studies [23–26] and we adapted it for use in individuals with TSC. The questionnaire included 36 questions relating to disease characteristics (e.g., genetics, affected organ systems, seizures, medications, and additional symptoms), healthcare resource use (e.g., healthcare visits, accidents, and emergency care), and social conditions. Paper questionnaires in German were sent to individuals with TSC between February and July 2019.

### Costing methods

The aim of this study was to calculate the specific genuine costs associated with TSC, rather than those associated with conditions unrelated to TSC. Therefore, we asked individuals in detail whether the medications, services, and other resources that were consumed were associated with particular organ manifestations of TSC. We evaluated costs using a bottom-up approach from the perspective of the statutory health insurer (“Gesetzliche Krankenversicherung” [GKV]) and society as a whole. The cost categories that were included in the analysis were direct health service costs, patients’ out-of-pocket (OOP) expenses, care grade allowances as approximation of informal care costs, and indirect costs. We evaluated these costs according to the German recommendations for performing health economic evaluations [27].

#### Direct health care costs

We obtained information regarding the direct health service costs from the literature and from standard reference sources for Germany, which were estimated as previously described [23, 26]. Direct health costs included specifically inpatient stays, outpatient visits, medicines (antiseizure drugs [ASDs], mTOR inhibitors, other prescription drugs, over-the-counter drugs, and emergency medications), medical aids, healthcare professional visits, emergency transportation, diagnostic studies, specific diets, individuals’ copayments, rehabilitation costs, private transport costs and copayments for therapies. We based drug costs on the Drug Prescription Report of 2019 (“Arzneiverordnungs-Report”) [28], which is an index of available medicines and their average prices in Germany. We standardized the costs of inpatient and outpatient care, specialist care, therapies, and diagnostic studies according to the method described by Bock et al. [29] and physician fee scales (Einheitlicher Bewertungsmaßstab) [30]. Costs were inflated to 2019 levels using the consumer price index for Germany and were expressed in both annual and 3-month terms in 2019 Euro.

#### Out-of-pocket expenses

All OOP expenses (copayments) that were reported were considered to be accounted for when supply-side cost estimates were calculated based on resource utilization (ancillary treatments, medical aids, healthcare professionals, and emergency transportation), and these OOP expenses were therefore not included in the calculation of total direct costs. We reported OOP expenses explicitly and added them to the total direct healthcare costs when supply-side utilization estimates were not available (care and supervision, healing agents, and diets) or when expenditures existed beyond the formal healthcare setting (alternative and occupational therapies and equipment costs).

#### Care grade allowances as approximation of informal care costs

We calculated the average care grade allowances [31] under the assumption that nursing services were provided by family members. Care grade allowances are the basis on which the German statutory care insurance pays care allowances. Care grade allowances are determined by the grade of necessary patient care, distinguished by levels 1–5 on the “Pflegegrade” scale. We used care grade costs as an approximation of informal care costs, and we separately reported any additional care costs reported by the respondents. While care grade allowances do not fully reflect the extent of informal care costs, we used them as a compromise between the goal of capturing a large set of individuals and the feasibility of assessing extent of informal care on an individual level.

#### Indirect costs

We calculated productivity losses due to TSC (days off, inability to work, reductions in working hours, or early retirement) using the human capital approach for patients of working age (i.e., below the age of 67). The mean annual gross wage of EUR 44,964 in 2019 [32] was used to calculate the productivity costs for each patient. For days taken off work, gross wages were calculated as EUR 215.14 per calendar day, and daily income was multiplied by total days off [24].

#### Grouping of questionnaire items

We collated some questionnaire items into groups when presenting the results. Specifically, the term “ancillary costs” includes physiotherapy, speech therapy, occupational therapy, acupuncture, hippotherapy and other ancillary costs. The term “healthcare professionals” includes neurologists, general practitioners (GPs), orthopedic surgeons, child psychiatrists, alternative medicine practitioners, homeopathy practitioners, dietitians and other specialists. The term “diagnostic studies” includes electroencephalography (EEG), blood tests, magnetic resonance imaging (MRI) or computed tomography (CT) scans, X-rays and other diagnostic studies.

For a detailed overview of the costing sources used, please refer to the Addtional file 1: Supplementary material.

### Statistical analysis

We conducted statistical analysis using IBM SPSS Statistics, version 26 (IBM Corp., Armonk, NY, USA). We summarized the variables of interest using the mean, median, and standard deviation (SD). For cost data, we calculated 95% confidence intervals (CI), using the bootstrap-corrected and accelerated (BCa) method with n = 2000 repetitions to estimate parameters robust to skewed distributions and outliers [33, 34]. Due to the small population of TSC and related statistical challenges [35, 36], we refrained from a power calculation or a predefined number of participants, and aimed to include all potential patients with TSC in Germany. We compared groups using adequate parametric and nonparametric tests after testing for the normality of distribution. The significance level was assumed at *p* < 0.05. We investigated the relationships between an individual’s clinical characteristics and TSC-related costs using multivariate linear regression using the BCa method with 2000 repetitions. Total direct, total indirect, and nursing care-level costs were regressed against a set of clinical variables, which we selected following univariate analysis and according to evidence presented by previous cost-of-illness studies examining TSC [16, 37, 38]. We tested all variables for interactions and collinearity. To identify independent predictors of costs, we performed standard multiple linear regression analysis using the bootstrapping technique and applied a Bonferroni correction for multiple testing.

## Results

### Demographic and clinical characteristics

One hundred and ninety-two adults with TSC completed the questionnaire. The mean participant age was 33.4 years (SD: 12.7 years; median: 31.0 years; range: 18.0–78.0 years), 51.6% (n = 99) were women. Among the respondents, TSC was diagnosed at a mean age of 10.4 years (SD: 14.9 years; median: 2.0 years; range: 0–66.0 years), and the first symptoms of TSC were noted at a mean age of 5.7 years (SD: 12.0 years; median: 0 years; range: 0–66.0 years). In three individuals (1.6%), a diagnosis of TSC was suspected before birth, based on ultrasound examinations. Pathogenic variants in *TSC1* were reported by 32 individuals (16.7%), and pathogenic variants in *TSC2* were reported by 48 individuals (25.0%, ratio 1:1.5). Three individuals (1.6%) suffered from a polycystic kidney disease with tuberous sclerosis (PKDTS) contiguous gene deletion syndrome.

Most individuals lived with others. Of those, 44 (22.9%) were married or in a relationship, and 84 (43.8%) lived with relatives. Less than half of individuals were either employed (n = 71, 37.0%) or participated in vocational training (n = 21, 10.9%). Further sociodemographic and clinical characteristics, including information on affected family members, are presented in Table 1. The majority of individuals suffered from a range of TSC organ manifestations. Disorders of the central nervous system were commonly reported, with 140 (72.9%) individuals reporting a diagnosis of epilepsy, 129 (67.2%) describing various structural brain disorders, and 97 (50.5%) indicating psychiatric disorders. Furthermore, 182 (94.8%) individuals reported skin manifestations, 142 (74%) described kidney and urinary tract disorders, and 97 (50.5%) indicated heart and circulatory system disorders. Additional details can be found in Table 2.

**Table 1:** Sociodemographic and clinical characteristics of participants (n = 192)

	All patients n = 192
Age in years^1^	33.4 ± 12.7
	Range
	18.0–78.0
Sex	% (n)
Male	48.4 (93)
Female	51.6 (99)
Age at first symptoms due to TSC^1^	5.7 ± 12.0
	Range
	0.0–66.0
Age at TSC diagnosis in years^1^	10.4 ± 14.9
	Range
	0.0–66.0
TSC diagnosis before birth by ultrasound	% (n)
No	96.9 (186)
Yes	1.6 (3)
Genetics	% (n)
*TSC1-gene*	16.7 (32)
*TSC2-gene*	25.0 (48)
*TSC2*/*PKD1* contiguous-gene	1.6 (3)
No genetic test	30.7 (59)
No genetic mutation	10.4 (20)
Unknown	15.6 (30)
Affected family members by TSC	% (n)
No	77.6 (149)
Yes	18.8 (36)
Mother affected (43.9 years)^2^	4.7 (9)
Father affected (46.3 years)^2^	3.6 (7)
Sibling affected (4.8 years)^2^	6.8 (13)
Own children affected (3.0 years)^2^	8.9 (17)
Number of own affected children	Mean
	1.4
Marital status	% (n)
Married/living in relationship	22.9 (44)
Divorced	1.6 (3)
Single, lives with relatives	43.8 (84)
Single, lives alone	29.7 (57)
Unknown/Other	2.1 (4)
School education	% (n)
< 12 years	42.7 (82)
> 12 years	20.8 (40)
Still going to school	4.7 (9)
No school graduation	30.2 (58)
Not answered	1.6 (3)
Highest job qualification	% (n)
Missing	42.2 (81)
Skilled (manual)	23.4 (45)
Office-based (nonmanual)	5.7 (11)
University degree	9.9 (19)
In training	8.3 (16)
Unknown/Other	10.4 (20)
Employment situation	% (n)
Employed	37.0 (71)
Vocational training	10.9 (21)
Unemployed	21.4 (41)
Homemaker/parental leave	1.0 (2)
Early retirement	9.9 (19)
Old-age pension	1.0 (2)
Unknown/Other	18.8 (36)

^1^Mean ± standard deviation

^2^Mean age at TSC diagnosis of affected family members

**Table 2:** Organ manifestations in individuals with TSC (n = 192)

	%	n
Epilepsy	72.9	140
Recurrent seizures	39.1	75
Seizure free > 1 year or no seizures	60.9	117
Structural brain disorders	67.2	129
Cortical tubers	49.0	94
SEGA^1^	37.5	72
Hydrocephalus	2.6	5
Psychiatric disorders	50.5	97
Heart and circulatory system	50.5	97
Hypertension	26.6	51
Rhabdomyomas	24.5	47
Arrhythmia	8.3	16
Kidney and urinary tract	74.0	142
Angiomyolipomas	59.4	114
Cysts	42.2	81
Chronic kidney dysfunction	12.5	24
Renal cell carcinoma	2.6	5
Skin manifestations	94.8	182
Angiofibromas	84.9	163
Hypomelanotic macules	57.3	110
Shagreen patches	48.4	93
Ungal/periungal fibromas	10.9	21
Skin tags	3.6	7
Café au lait spots	2.6	5
Lymphangioleiomyomatosis	11.5	22
Other disorders	39.1	75
Iris or retinal hamartomas/astrocytomas and other eye disorders	28.6	55
Angiomyolipomas in other organ systems^2^	14.1	27
Cysts in other organ systems^2^	13.0	25

^1^Subependymal giant cell astrocytoma

^2^Hormone system, Thyroid, Gastrointestinal, Liver, Spleen, Pancreas

### Direct costs

**Fig. 1:**
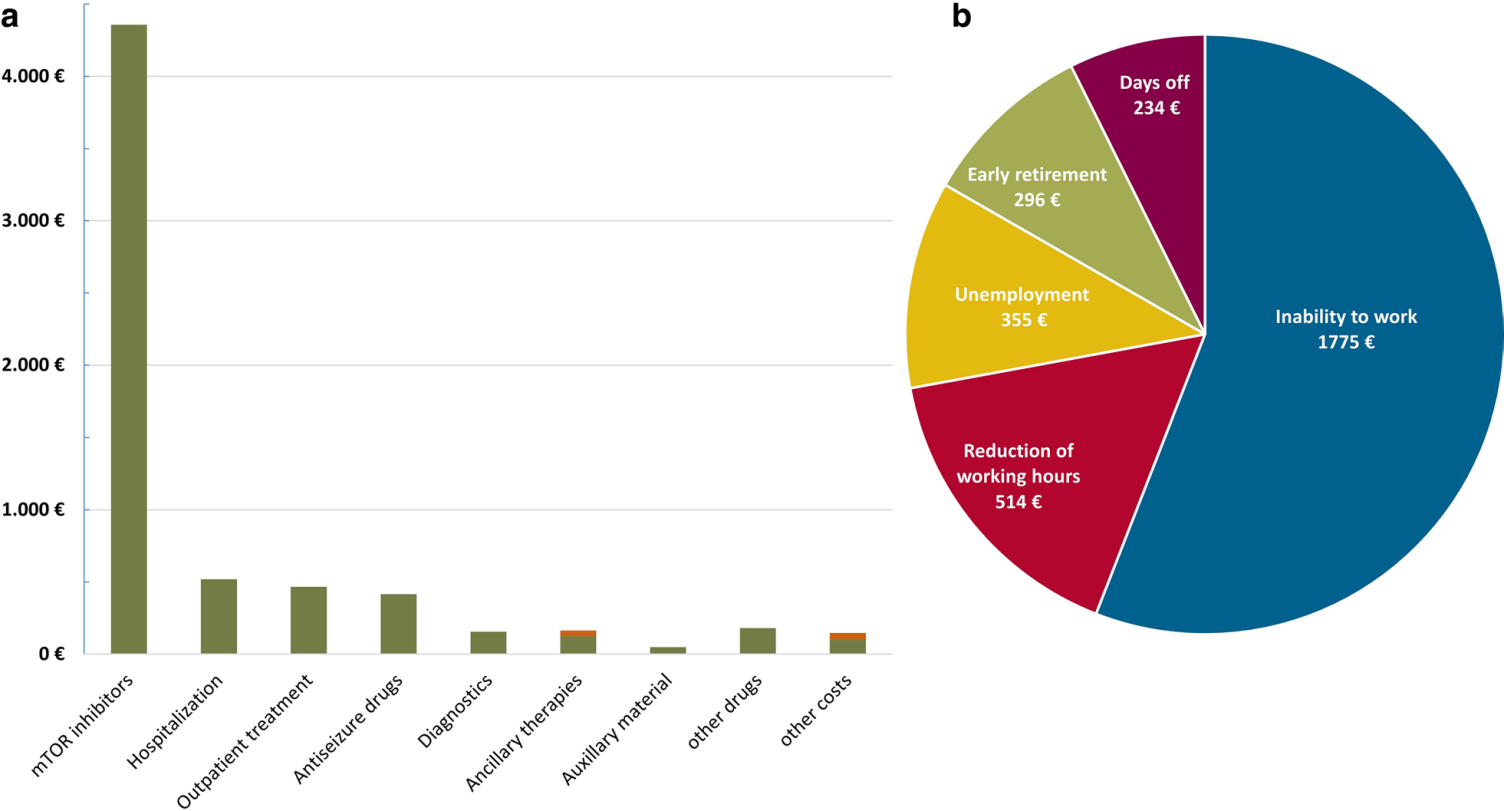
Breakdown of total direct costs (**a**), with copayments in orange, per patient over 3 months, and total indirect costs over 3 months (**b**)

Mean total direct costs were calculated at EUR 6452 (median EUR 1920; 95% CI EUR 5533–7422) per study participant for the 3-month study period, and details are presented in Table 3 and Fig. 1. Direct medical costs were primarily associated with the costs of drug treatments (76.8% of total direct costs; mean EUR 4953 per 3 months; median EUR 573; 95% CI EUR 4087–5876), and hospitalization (8.0% of total direct costs; mean EUR 518; median EUR 0; 95% CI EUR 312–750).

**Table 3:** Direct costs for the 3-month study period for the total patient group (n = 192; in 2019 Euro)

Cost components	Mean costs	SD^1^	Minimum	Median	Maximum	95% CI	% of total direct costs	Estimated annual direct costs^2^
Total direct costs	6452	7584	0	1920	29,182	5533; 7422	100	25,808
Medication (n = 165)	4953	6854	0	573	28,224	4087; 5876	76.8	19,812
mTOR inhibitors* (n = 71)	4358	6520	0	0	25,273	3448; 5342	67.5	17,432
Antiseizure drugs (ASDs) (n = 123)	415	1962	0	104	26,538	239; 706	6.4	1660
Other prescription drugs (n = 104)	132	385	0	8	2606	84; 186	2.0	528
OTC drugs and supplements (n = 70)	41	100	0	0	700	29; 54	0.6	164
Emergency medication/medication on demand (n = 42)	7	36	0	0	347	3; 13	0.1	28
Hospitalization (n = 23)	518	1691	0	0	11,487	312; 750	8.0	2072
Outpatient treatment (n = 157)	467	1156	0	194	15,097	352; 626	7.2	1868
Diagnostics (n = 140)	155	242	0	44	1691	124; 192	2.4	620
Ancillary therapies (n = 54)	125	307	0	0	2120	84; 174	1.9	500
Auxillary material (n = 14)	49	253	0	0	2235	18; 87	0.8	196
Rehabilitation (n = 2)	40	410	0	0	4983	0; 92	0.6	160
Emergency service use (n = 9)	44	217	0	0	1800	19; 75	0.7	176
Specific diets (n = 3)	9	97	0	0	1200	0; 23	0.1	36
Transport costs (n = 25)	5	17	0	0	130	3; 7	0.1	20
Co-payments for therapies (n = 17)	39	177	0	0	1400	19; 64	0.6	156
Other co-payments (n = 45)	48	183	0	0	1700	27; 75	0.7	192

95% CI = 95% Confidence interval using the bootstrap bias corrected and accelerated method

^1^Standard deviation, ^2^Estimation based on the mean costs in three months multiplied by four

*Everolimus n = 69, Sirolimus n = 2, OTC = over-the-counter

The largest medication costs were those due to mTOR inhibitors (everolimus, n = 69; sirolimus, n = 2), with a mean of EUR 4358 per 3 months (67.5% of total direct costs; median EUR 0; 95% CI EUR 3448–5342). mTOR inhibitor costs were higher than those associated with ASDs, which were on average EUR 415 (6.4% of total direct costs; median EUR 104; 95% CI EUR 239–706). Individuals used on average 1.9 ASDs (SD: 0.8; median 2: range 1–4). The five most frequently prescribed ASDs were lamotrigine (n = 51; 26.6%), valproate (n = 46, 24.0%), oxcarbazepine (n = 32; 16.7%), levetiracetam (n = 25; 13.0%), and lacosamide (n = 12; 6.3%). Monotherapy with ASDs was prescribed to 24.0% (n = 46) of all participants, which was associated with significantly lower costs than polytherapy with two, three, or more ASDs (each *p* < 0.001). The detailed costs and daily dosages reported for different ASDs are listed in Table 4.

**Table 4:** Prescription patterns and costs of antiseizure drugs for the 3-month study period (in 2019 Euro)

Medication costs	n	Mean costs per 3 months	SD^1^	Minimum	Median	Maximum	95% CI	p-value^2^
All patients	192	€ 415	1962	€ 0	€ 104	€ 26,538	239; 706	
No ASDs (35.9%)	69	0						
Monotherapy (24.0%)	46	€ 222	400	€ 15	€ 125	€ 2613	136; 354	< 0.001^3^
2 ASDs (26.0%)	50	€ 327	317	€ 50	€ 238	€ 1405	250; 415	< 0.010^4^
≥ 3 ASDs (14.1%)	27	€ 1965	4979	€ 144	€ 962	€ 26,538	826, 4016	< 0.001^5^

Prescribed medication	n	Mean daily dose	SD^1^	Minimum	Median	Maximum	Mean costs per 3 months	SD^1^
Lamotrigine (26.6%)	51	362 mg	373 mg	50 mg	300 mg	2500 mg	€ 90	93
Valproate (24.0%)	46	1355 mg	465 mg	450 mg	1250 mg	2300 mg	€ 62	21
Oxcarbazepine (16.7%)	32	1617 mg	708 mg	150 mg	1800 mg	3600 mg	€ 226	99
Levetiracetam (13.0%)	25	2400 mg	1090 mg	750 mg	2250 mg	4500 mg	€ 161	73
Lacosamide (6.3%)	12	410 mg	283 mg	100 mg	350 mg	1200 mg	€ 894	616
Topiramate (3.6%)	7	225 mg	56 mg	150 mg	200 mg	300 mg	€ 149	37
Zonisamide (3.6%)	7	657 mg	450 mg	300 mg	500 mg	1600 mg	€ 1087	745
Carbamazepine (3.1%)	6	700 mg	490 mg	300 mg	500 mg	1600 mg	€ 35	24
Perampanel (3.1%)	6	5 mg	2 mg	2 mg	6 mg	8 mg	€ 308	120
Brivaracetam (2.6%)	5	220 mg	76 mg	150 mg	200 mg	350 mg	€ 508	175
Sulthiame (2.6%)	5	530 mg	470 mg	100 mg	450 mg	1250 mg	€ 320	284
Vigabatrine (2.6%)	5	2200 mg	758 mg	1000 mg	2500 mg	3000 mg	€ 397	137
Phenytoin (2.1%)	4	331 mg	85 mg	250 mg	313 mg	450 mg	€ 26	7
Primidone (2.1%)	4	750 mg	451 mg	375 mg	625 mg	1250 mg	€ 38	23
Phenobarbital (2.1%)	4	141 mg	106 mg	10 mg	163 mg	230 mg	€ 62	47
Rufinamid (1.6%)	3	1267 mg	702 mg	600 mg	1200 mg	2000 mg	€ 759	421
Clobazame (1.6%)	3	25 mg	13 mg	15 mg	20 mg	40 mg	€ 68	36
Gabapentin (1.6%)	3	967 mg	1250 mg	100 mg	400 mg	2400 mg	€ 78	101
Other ASDs* (5.7%)	11							

^1^ Standard deviation, 95% CI = 95% Confidence interval using the bootstrap bias corrected and accelerated method

^2^Mann-Whitney-U-test; ASD = antiseizure drug; ^3^Monotherapy vs. ≥ 3 ASDs, ^4^Monotherapy vs. 2 ASDs^, 5^2 ASDs vs ≥ 3 ASDs,

*(Cannabidiol n = 2, Clonazepam n = 2, Ethosuximide n = 2, Lorazepam n = 2, Mesuximide n = 1, Pregabalin n = 2)

In total, 23 (12.0%) individuals reported at least one TSC-related hospital admission during the 3-month study period. Overall, 29 admissions were reported, with a mean length of stay of 5.5 days (SD: 3.6; median: 5 days; range: 1–14 days). Epilepsy and seizures resulted in eight admissions, whereas six admissions were associated with diagnostic procedures, four admissions were due to pneumothorax, three were related to operations concerning AML in the kidneys and other organs. A further two admissions were due to adverse reactions to everolimus intake and two were associated with facial skin treatments. Four admissions had other TSC-related reasons.

Ancillary treatments, such as occupational therapy, physiotherapy, and speech therapy, were prescribed to 54 participants (28%), and were associated with an average cost of EUR 125 per 3 months, which comprised 1.9% of total direct costs (median: EUR 0; 95% CI: EUR 84–174). In addition, families directly paid EUR 39 in therapy-related costs during the 3-month study period.

### Care needs and care grade as approximators of informal care costs

Fifty percent (n = 97) of individuals were categorized as requiring care grade levels I to V, based on the “Pflegebedürftigkeit” scale: 2.6% as level I (‘low impairment of independence’); 9.9% as level II (‘significant need for care’); 11.5% as level III (‘heavy need for care’); 13.5% as level IV (‘most difficult to care for’); and 13% as level V (‘most difficult to care for and special demands regarding nursing care’). Two individuals did not meet the level I–V criteria but were still in need of care according to their caregivers, and 48.4% of individuals denied being in need of care. The mean approximate costs for nursing care were EUR 929 (median: EUR 0; 95% CI: EUR 780–1083) over each 3-month period, or EUR 3716 annually, assuming that care is provided by family members. Patient’s caregivers reported that they had paid additional costs for care, with a mean of EUR 24 (median; EUR 0; 95% CI: EUR 10–41). Furthermore, they paid for supervision, with a mean of EUR 48 (median: EUR 0; 95% CI: EUR 22–79) per 3-month period. Further informal care costs that were neither reflected in the care grade allowance nor perceived by caregivers are inevitably not represented in our approximation of informal care costs. In total, 124 individuals (64.6%) had a handicapped ID, indicating a degree of disability between 70 and 100%.

### Indirect (productivity) costs

The estimation of mean indirect costs was based only on questionnaire responses from patients of working age, younger than 67 years (n = 190). The mean total indirect costs were EUR 3174 (median: EUR 0; 95% CI: 2503–3840) over three months or EUR 12,696 annually. The main contributor to indirect costs (n = 30) was the inability to work due to intellectual disability, epilepsy, or kidney disorders (mean: EUR 1775; median: EUR 0; 95% CI: EUR 1183–2367). Furthermore, 19 individuals were only able to work part-time, which was associated with a mean estimated cost of EUR 514 ± 1762 per 3 months (median: EUR 0; 95% CI: EUR 283–792). Twenty-eight individuals reported missing days from work during the last three months due to TSC-related causes (mean: EUR 234; median: EUR 0; 95% CI: EUR 115–382), and six individuals were unemployed (mean: EUR 355; median: EUR 0; 95% CI: EUR 118–651). Five individuals reported retiring prematurely (mean: EUR 296; median: EUR 0; 95% CI: EUR 59–592). The details of indirect productivity costs can be found in Table 5 and Fig. 1. The mean duration of work absenteeism was 7.6 ± 10.7 days (range: 1–50 days) per 3 months.

**Table 5:** Indirect costs for individuals with TSC during the 3-month study period (in 2019 Euro)

Indirect costs components	n^1^	Mean costs	SD^2^	Minimum	Median	Maximum	95% CI	Estimated annual costs^3^
Total indirect costs (< 67 y)	86	3174	4703	0	0	11,241	2503; 3840	12,696
Inability to work	30	1775	4110	0	0	11,241	1183; 2367	7100
Reduction of working hours	19	514	1762	0	0	9695	283; 792	2056
Unemployment	6	355	1971	0	0	11,241	118; 651	1420
Early retirement	5	296	1804	0	0	11,241	59; 592	1184
Days off	28	234	1050	0	0	10,750	115; 382	936

95% CI = 95% Confidence interval using the bootstrap bias corrected and accelerated method

^1^Patients of working age (n = 190), ^2^Standard deviation, ^3^Estimation based on the mean costs in three months multiplied by four

### Cost drivers of direct, indirect, and approximated informal (nursing) care costs

To identify potential cost drivers, we performed univariate analyses for total direct, total indirect, and nursing care costs and a number of demographic and clinical characteristics. For details, please refer to Table 6. In the univariate analyses, younger age, the use of mTOR inhibitors, polytherapy with two or more ASDs, recurrent seizures, all TSC manifestation categories, the total number of TSC manifestations, and the level of disability were associated with higher direct costs. Lung manifestations (lymphangioleiomyomatosis), the total number of TSC manifestations, and disability were associated with higher indirect costs, whereas younger age, polytherapy with two or more ASDs, recurrent seizures, the TSC manifestations of epilepsy, structural brain disorders, psychiatric disorders, and skin manifestations, the total number of manifestations, and disability were associated with increased nursing care costs. Overall, total direct, indirect, and nursing costs increased with the number of affected organ systems (Table 6).

**Table 6:** Univariate and multivariate analysis of cost-driving factors for total direct, total indirect, and nursing care-level costs (3-month period; in 2019 Euro)

	n	Total direct costs in €	Median	SD	p-value^§^	Total indirect costs in €**	Median	SD	p-value^§^	Nursing care level costs in €	Median	SD	p-value^§^
Gender					0.321				0.088				0.082
Male	93	6173	1527	8156		2796	0	4687		1079	948	1102	
Female	99	6714	2722	7036		3529	538	4714		788	0	1016	
Age					0.012*				0.062*^#^				0.003*
18–29 years	90	8067	3460	8419		2171	0	4117		1084	948	1081	
30–39 years	54	5849	1646	6700		3978	323	5081		1072	948	1072	
40 years and above	48	4102	1350	6154		4192	430	5011		477	0	911	
Number of antiseizure drugs					< 0.001				0.057				< 0.001
≥ 2	77	9237	9743	8639		4054	430	5131		1461	1635	1004	
0—1	115	4587	1224	6155		2574	0	4308		573	0	955	
mTOR inhibitors intake					< 0.001^#^				0.510				0.250
Yes	71	14,473	13,100	5641		3335	0	4729		1041	948	1085	
No	121	1746	712	3548		3077	0	4705		863	0	1053	
Seizures					< 0.001^#^				0.105				< 0.001
Recurrent seizures	75	9082	5440	8930		3859	323	5061		1530	1635	1055	
Seizure free > 1 year or no seizures	117	4729	1401	5997		2717	0	4412		535	0	875	
Epilepsy					0.001				0.904				< 0.001^#^
Yes (72.9%)	140	7195	2184	8106		3284	0	4866		1249	1635	1072	
No (27.1%)	52	4451	1257	5545		2866	0	4242		68	0	288	
Structural brain disorders					0.003				0.889				0.014
Yes (67.2%)	129	7353	2343	8041		3106	0	4681		1059	948	1099	
No (32.8%)	63	4607	1307	6211		3318	0	4784		663	0	947	
Psychiatric disorders					< 0.001				0.260				< 0.001^#^
Yes (50.5%)	97	8034	2982	8083		3741	0	5110		1521	1635	1037	
No (49.5%)	95	4837	1199	6703		2583	0	4182		325	0	693	
Heart and circulatory manifestations					< 0.001				0.558				0.170
Yes (50.5%)	97	8180	6568	7764		3229	0	4719		1032	948	1113	
No (49.5%)	95	4687	1307	7007		3118	0	4710		823	0	1010	
Kidney and urinary tract manifestations					< 0.001				0.248				0.317
Yes (74.0%)	142	7311	3357	7419		3381	0	4768		973	474	1078	
No (26.0%)	50	4013	614	7591		2594	0	4511		805	0	1030	
Skin manifestations					0.009				0.296				0.004
Yes (94.8%)	182	6704	2112	7669		3207	0	4702		980	948	1072	
No (5.2%)	10	1873	380	3666		2498	0	4957		0	0	0	
Lung manifestation					0.030				0.014				0.139
Yes (11.5%)	22	8894	7646	7928		5249	2588	5220		598	0	912	
No (88.5%)	170	6136	1667	7505		2902	0	4578		972	0	1079	
Other disorders					0.013				0.116				0.959
Yes (39.1%)	75	7856	7315	7352		3711	430	4982		938	0	1070	
No (60.9%)	117	5552	1460	7625		2823	0	4499		923	0	1067	
Total Disorders					< 0.001*				0.031*				< 0.001*
1–3 Manifestations (27.1%)	52	2652	331	5369		1982	0	3890		361	0	786	
4 Manifestations (20.3%)	39	6488	1980	7796		2676	0	4299		652	0	922	
5 Manifestations (19.8%)	38	6353	1739	7158		4816	1075	5345		1212	1292	1065	
6 Manifestations (19.8%)	38	8080	6861	8022		3169	215	4770		1193	948	1070	
7–8 Manifestations (13.0%)	25	11,977	15,151	7402		3844	0	5176		1709	2184	1083	
Level of disability					< 0.001				0.001^#^				< 0.001^#^
None or ≤ 60%	68	4395	496	6823		1483	0	3085		110	0	470	
70–100%	124	7580	2641	7768		4074	323	5158		1378	1635	1034	

^§^Mann–Whitney-U-test; *Kruskal–Wallis-test; **for indirect costs only indivuals of woking age (n = 190) were considered; SD = standard deviation; ^#^significant predictor in multivariate analysis

Multiple linear regression analyses revealed that the use of mTOR inhibitors independently predicted a 3-month direct cost increase of 12,069 Euro (BCa-corrected B 12,068.85, BCa-corrected standard error [SE] B 836.28, β 0.770, *p* < 0.001), and persistent seizures predicted a 3-month direct cost increase of 2113 Euro (B 2113.29, SE B 651.13, β 0.137, *p* < 0.001). Applying a Bonferroni correction for twelve comparisons, the threshold for the p-value was set at 0.00417, and the mTOR inhibitor use and persistent seizures were able to explain 71% (*R*^2^, F (12, 179) = 37.09, F sig.) of the total direct cost variance. Older age and disability were independent cost-driving factors for total indirect costs, with disability predicting a 3-month indirect cost increase of 2131 Euro (B 2131.48, SE B 693.90, β 0.216, *p* = 0.004) and older age of 1220 Euro per 3 months (B 1220.05, SE B 391.20, β 0.212, *p* = 0.003). The significant factors together explained 13% of the indirect cost variance (corrected *p* < 0.0125; *R*^2^ = 13%, F (5, 184) = 5.67, F sig.). Epilepsy, psychiatric diseases, and disability were independent cost drivers for approximate informal (nursing) care costs, with relatively similar cost-driving effects. Disability predicted a 3-month nursing-cost increase of 622 Euro (B 622.02, SE B 160.86, β 0.280, *p* < 0.001), psychiatric diseases of 599 Euro (B 599.45, SE B 149.67, β 0.282, *p* < 0.001), and epilepsy of 528 Euro (B 528.27, SE B 138.67, β 0.221, *p* = 0.001). Together, these significant variables (corrected *p* < 0.00625) were able to explain 51% of the 3-month nursing care cost variance (*R*^2^ = 51%, F (9, 182) = 20.98, F sig.).

## Discussion

This detailed, multicenter, COI study is based on a large sample of 192 adult individuals with TSC within a single healthcare system and contributes important new information about the direct and indirect costs and related cost drivers associated with TSC in Europe. To enable comparisons with other COI studies, we aimed to capture the most comprehensive set of cost items related to epilepsy and other TSC organ manifestations [4, 39]. Previous studies have reported direct cost estimates for individuals with TSC in Europe [15, 16, 40, 41], North America [9, 11, 37, 38, 42–44], and Asia [45], but none of these previous studies have provided indirect cost estimates for adults affected by TSC or examined the cost drivers of indirect and nursing care costs [4].

### Direct costs and related cost drivers

Our study highlights the substantial direct costs incurred by individuals with TSC. Medication was the largest single component of direct costs and associated with an estimated annual direct cost of EUR 25,808. The highest medication costs were due to mTOR inhibitors (annual costs of EUR 17,432), which were used by 37% (71/192) of the individuals in this study. mTOR inhibitor use was identified as an independent cost-driver in the multivariate analysis. This finding is somewhat expected, given the currently high price of mTOR inhibitors. Everolimus, the mTOR inhibitor overwhelmingly used by individuals in this study, was first given conditional marketing authorization as an orphan drug by the European Medicines Agency (EMA) in 2011 for TSC-associated SEGA and in 2012 for TSC-associated renal AML [46], followed by an extension of indication to epilepsy refractory to ASD in 2017. Due to the time point of this study, the long-term cost-effectiveness of this currently costly drug remains to be evaluated [47]. The possibility of avoiding potentially costly TSC consequences, such as resection surgery for SEGA or epilepsy, cerebral shunt placement, and AML-associated renal bleeding following everolimus may balance favorably against the unwanted treatment effects of mTOR inhibitors, and everolimus may emerge as a cost-effective treatment option. The costs associated with the use of mTOR inhibitors will also likely decrease in the future due to the availability of generic formulations.

Interestingly, gender differences were not associated with differences in TSC-related costs. Women with TSC are known to be more likely to develop AML, and AML in women tends to be larger and require more interventions [10]. Women are also almost exclusively affected by pulmonary manifestations of TSC, which have been demonstrated to incur high direct costs [11, 15]. The findings in the present study may be due to the relative rarity of severe LAM complications, which typically occur only with increasing age. However, we did record four hospital admissions among our cohort for pneumothorax, a known complication of LAM, within the short evaluation period of three months. An alternative explanation is that the salient contributions of mTOR inhibitor therapy to overall costs, which exceeded inpatient treatment costs, may have masked gender differences in our cohort. Young adults (18–29 years) with TSC incurred higher costs than older individuals did, which is likely associated with the performance of a larger proportion of diagnostic procedures and the increasing use of mTOR inhibitors among younger patients.

In adults, renal manifestations of TSC are more common than in children. The most common types of renal manifestations, including AML and renal cysts, tend to appear first during adolescence and grow during adulthood. In our study, a similar proportion of individuals reported AML as in the TOSCA cohort [10], and renal manifestations were a significant factor for direct costs in the univariate analysis. Interestingly, in the multivariate analysis, only recurring seizures remained an independent clinical cost driver, and no single other TSC manifestation category was identified as an independent cost driver, which may be due to the higher direct costs associated with recurrent seizures compared with all of the other seven clinical categories, which were all associated with similar direct costs. This finding supports the known severe burden of illness of ongoing epilepsy in TSC, particularly because a relevant share of individuals experience pharmacorefractory epilepsy [48]. The high direct costs are in line with those reported for other rare developmental and epileptic encephalopathies like Dravet syndrome [49] or Lennox-Gastaut-syndrome [50]. This finding further highlights the need for ongoing identification of epilepsy surgical candidates among those with TSC and epilepsy, new emerging therapies such like the MR-guided laser interstitial thermal therapy might help to increase the suitability of patients for a surgical treatment [51]. Generally, the results of this study indicate that the management of TSC might result in high direct costs that exceed the costs incurred by all-cause epilepsy patients [26]. Our multivariate analysis model was able to account for 71% of the total variance in directs costs, suggesting that mTOR inhibitor use together with recurring seizures can explain a relevant share of the direct cost components among TSC patients in Germany. Similar to results reported for the United Kingdom [15], direct costs increased with the number of TSC manifestations. Although this finding was expected due to the complexity of TSC, which necessitates surveillance and treatment for most manifestations, this finding further demonstrated the need for systemic causal treatment and integrated, streamlined care, such as by specialized TSC centers.

Cannabidiol (CBD) is a new treatment option for drug-resistant seizures associated with TSC, it has recently been approved by the United States Federal Drug Administration (FDA) and the EMA. CBD has shown promising results in a randomized-controlled study published recently [52]. Since our study preceded the approval of CBD for TSC in the European Union, only two patients were treated with CBD in our cohort and the influence of CBD on the direct cost is negligible in our results.

Most cost categories were heavily skewed due to the clinical heterogeneity among adult individuals with TSC. While a few patients required significant health care resources, many were only mildly affected. In our study, the ratio of patients diagnosed with *TSC1* vs. *TSC2* mutations was slightly higher than anticipated from past study findings [12, 53]. Because individuals with *TSC2* mutations tend to be more severely affected, especially by neuropsychiatric manifestations and epilepsy, an even higher COI can be presumed among populations with higher shares of *TSC2* mutations. However, we caution that a large proportion of the patients in this study did not report any genetic test results.

### Indirect costs and related cost drivers

To our knowledge, this is the first study to report the indirect costs of TSC. Nearly half of individuals (86/192, 46%) who participated in this study reported productivity losses. Adult individuals with TSC in our study incurred substantial indirect annual costs, equal to EUR 12,696 per year, with the largest share due to the inability to work (EUR 7100). Approximately two-thirds (132/192, 67%) of individuals in our study were able to work without impairment (excluding extra days off due to TSC). The indirect costs reported in our study are broadly similar to those reported for other rare neurological diseases, such as spinal muscular atrophy [54] or Becker’s muscular dystrophy [55], but were substantially higher than those reported among individuals with all-cause epilepsy [24]. Unlike direct costs, only a few clinical or demographic categories were associated with increased indirect costs on univariate analysis. We identified both the manifestation burden and the level of disability as associated with increased indirect costs. LAM also emerged as a variable significantly associated with higher indirect costs, indicating that while rare overall, LAM may play an outsize role in affected individuals due to its associated severe impairments.

Incomplete participation due to part-time work, unemployment, and early retirement was common even among those who were able to participate in the primary work market. Additionally, TSC impairs participation in education, as demonstrated by the high proportion of individuals that did not report any job qualifications (42.2%) and that did not graduate from school (30.2%). In addition, half of patients required care on the care grade allowance scale. Most clinical variables were not independently associated with higher approximated informal (nursing) care costs in the multivariate analysis, e.g. heart manifestations (which was primarily arterial hypertension among this adult cohort), renal manifestations, and lung manifestations. These types of clinical manifestations are typically intermittent, as with AML bleeding, or mostly do not necessitate nursing care, such as renal or pulmonary insufficiency. In contrast, epilepsy, neuropsychiatric manifestations, and disability were independent cost drivers that frequently required nursing care. We must caution that our approach of assessing informal care costs from allowances according to care grades necessarily misses all intangible informal costs that were not perceived by the caregiver as further costs and thus not specifically reported. This includes e.g. losses of caregiver productivity due to providing informal (= not by a health professional) care. True informal care costs are thus most likely higher than in our approximation.

### Comparison with earlier studies

The direct costs identified in our study are broadly comparable to those reported by other recent studies (see Table 7 for a comparison of costs). However, costs in our cohort were both higher than the annual direct costs reported by studies from the UK (GBP 4227–5054 per year) [15, 16, 41] and lower than the costs reported by two studies from the US (USD 8543–85,397 per year) [37, 38]. The direct costs associated with ASD were lower in our study than those reported in previous studies, despite the specific drugs that were reported being similar [44]. This finding likely reflects lower medication costs in Germany due to price negotiations between statutory health insurers and drug manufacturers in recent years [56]. Importantly, this finding of lower costs was not true for mTOR inhibitors, which incurred higher costs than were reported by another study from the US [44]. The costs associated with hospitalizations and outpatient treatment were lower in our study than in earlier studies. Interestingly, ancillary therapies represented a smaller share of total direct costs than were reported in another study that explicitly reported this variable [43], which is likely due to differences in the reimbursement policies between varying healthcare systems. In general, direct comparisons to studies from different settings or countries are difficult because due to differing definitions, policies, measurements and other factors. To date, only one other study has analyzed productivity losses in adults with TSC [43]. Interestingly, although this study was performed in a different country, activity impairment was similar, with approximately one-third of adult individuals with TSC reporting not fully participating in the work market.

**Table 7:** Studies on direct and indirect costs among adults with TSC

	Zöllner et al. current study	Betts et al. [44]	Chu et al. [45]	Skalicky et al. [43]	Song et al. [37]	Shepherd et al.[16]*	Kingswood et al. [15]*	Kingswood et al. [41]*	Wilson et al. [42]	Sun et al. [38]	Vekeman et al. [40]	Kristof et al. [11]
Study design	Multicenter, p	Multicenter, r	Multicenter, r	Multicenter, p	Multicenter, r	Multicenter, r	Multicenter, r	Multicenter, r	Multicenter, r	Multicenter, r	Monocenter, r	Multicenter, r
Costing year	2019	2019	2017	2012	2013	2014	2014	2014	n.r	2010	2012	2011
Country (city)	Germany	US	Hong Kong	US	US	UK	UK	UK	US	US	Netherlands(Utrecht)	Canada(Quebec)
Group	All TSC	TSC and epilepsy	All TSC	All TSC	TSC and AML	TSC and epilepsy	All TSC	TSC and kidneys	All TSC	TSC and SEGA surgery	TSC and kidneys	All TSC (especially LAM)
Number of patients	192	2028	284	430	487	209	286	79	5655	47	369	1004
Study population	A	C & A	C & A	A	A	C & A	C & A	C & A	C & A	C & A	C & A	A
Patients with epilepsy	72.9%	100%	71.3%	n.r	n.r	100%	n.r	n. r	41.2%	91%	n.r	7.8%^#^
Age in years (median)	18.0–78.0 (31.0)	Mean 25.3	0.45–89.9 (27.2)	19.0–83.0 (36.5)	Mean 36.9	Mean 26.8	Mean 31.5	Mean 38.3	Mean 22.3	Mean 11.6	Mean 42.8 ^7^	Mean 39.5
Patients with AED intake	64.1%	89.5%	n. r	n.r	n.r	88%	42.7%	68.4%	n.r	n.r	n.r	18.3%
Patients with mTOR-inhibitor intake	37.0%	10%	16.5%	n.r	8%	n.r	n.r	n.r	n.r	n.r	n.r	13.4% ^3^

	Mean PPPY	Mean PPPY	Mean PPPY	Median PPPY	Mean PPPY	Mean PPPY	Mean PPPY	Mean PPPY	Median	Mean PPPY	Mean PPPY	Mean PPPY
Total direct costs	EUR 6452	n.r	n.r	n.r	USD 32,858–48,499 ^4^	GBP 4778 ^5^	GBP 4227 ^5^	GBP 5054 ^5^	n.r	USD 8543–85,397 ^6^	EUR 1275–31,916 ^8^	n.r
Medication	EUR 4953	USD 18,836	n.r	USD 7200 ^1, 3^	USD 3103–4770 ^4^	*no specific amount*	GBP 595 ^5^ *(only primary care)*	GBP 869 ^5^ *(only primary care)*	n.r	USD 1300–2338 ^6^	EUR 429–1508 ^8^	n.r
* AED*	EUR 415	USD 12,866	n.r	n.r	n.r	n.r	n.r	n.r	n.r	n.r	n.r	n.r
* mTOR inhibitors*	EUR 4358	USD 4028	n.r	n.r	n.r	n.r	n.r	n.r	n.r	n.r	n.r	n.r
Hospitalization	EUR 518	USD 2106	USD 5819 ^1^	USD 2650 ^2, 3^	USD 15,390–11,787 ^4^	*no specific amount*	GBP 2181 ^5^	GBP 2350 ^5^	USD 14,807	USD 3770–71,562 ^6^	n.r	n.r
Ancillary therapies	EUR 125	n.r	n.r	USD 8160 ^2, 3^	n.r	n.r	n.r	n.r	n.r	n.r	n.r	n.r
Outpatient treatment	EUR 467	USD 13,455	USD 1414 ^1^	USD 500 ^2^	USD 13,639–31,158 ^4^	*no specific amount*	GBP 645 ^5^	GBP 690 ^5^	n.r	USD 3473–11,497 ^6^	n.r	CAD 513
ER visists	n.r	USD 1535	USD 116 ^1^	USD 500 ^2^	USD 725–785 ^4^	n.r	n.r	n.r	n.r	n.r	n.r	n.r

	Mean PPPY											
Total indirect costs	EUR 3174	n.r	n.r	n.r	n.r	n.r	n.r	n.r	n.r	n.r	n.r	n.r
Inability to work	EUR 1775	n.r	n.r	n.r	n.r	n.r	n.r	n.r	n.r	n.r	n.r	n.r
Part time work	EUR 514	n.r	n.r	n.r	n.r	n.r	n.r	n.r	n.r	n.r	n.r	n.r
Unemployment	EUR 355	n.r	n.r	n.r	n.r	n.r	n.r	n.r	n.r	n.r	n.r	n.r
Early retirement	EUR 296	n.r	n.r	n.r	n.r	n.r	n.r	n.r	n.r	n.r	n.r	n.r
Days off	EUR 234	n.r	n.r	n.r	n.r	n.r	n.r	n.r	n.r	n.r	n.r	n.r

p = prospective; r = retrospective; C = Children; A = Adults; n.r. = not reported; PPPY = per person/per year; *same study cohort; ^#^referring to epilepsy as discharge diagnosis

^1^10% of actual expenses, government subsidized more than 90%; ^2^ "out-of-pocket" direct spending; ^3^ calculated

^4^The first amount is from commercial cohort, the second one from medicaid cohort

^5^Calculated for one year, original cost figure given for a 3 year period, excluding GP administartion encounters

^6^From SEGA pre-surgery to post-surgery period; ^7^ calculated across all CKD stages

^8^The first amount is from CKD stage 1, the second one from CKD stage 5, overall mean PPPY costs for AML: EUR 1451–3243

In terms of the use of healthcare resources, our results appear to be in line with other COI studies, particularly hospitalization frequency and the use of ASDs [9, 11, 15, 16, 37, 42, 44, 45]. Most studies could not evaluate mTOR inhibitor use because the periods of data inclusion preceded their authorization. In our study, more patients (37%) used mTOR inhibitors than in three other recent studies (8–16.5%) [37, 44, 45].

A particular contribution of this study is the collection of data regarding the indirect costs and the nursing requirements, which were measured by the care grade allowances. Nursing-associated costs were identified as an important cost component, associated with annual expenditures of EUR 3716, reinforcing the importance of different organ manifestations and seizure-related costs, as were reported by Skalicky et al. [43].

### Limitations

The limitations associated with the questionnaire used in this study include the potential for recall bias regarding three-month-old events, which might result in incomplete and underestimated costs. Furthermore, although the sample consisted of individuals recruited from a variety of sources (multiple clinics and centers across Germany and through the patient advocacy group), we do not know whether the included sample is representative of individuals with TSC in Germany owing to the rarity of TSC. Only two individuals older than 67 years of age were included in this study, which may indicate the limited access to specialized care among this vulnerable group. In addition, the analysis of cost drivers should be interpreted with caution given the limited sample size. However, the significance that the number of organ manifestations had on COI in the current study suggests some common ground with earlier studies, which also identified the number of organ manifestations as cost driver [16]. In addition, the skewness identified in the cost calculations should be noted, as disparities were noted between the mean and median costs. A limitation is the incomplete capturing of informal care costs, as we based these only on allowances paid to the individual for support of caregivers, and services that were paid out-of-pocket by the patients or caregivers. Further intangible and likely substantial informal care costs such as work productivity loss of caregivers is explicitly exempt from our analysis. Another limitation of the study was due to the calculation of indirect costs using the human capital approach. The study was performed in 2019 in a situation of nearly full employment in the general population before the onset of the Corona virus disease (COVID-19) pandemic, however indirect costs may not exactly reflect the burden on society and may be overestimated [57]. However, due to the limitations of the friction cost approach [58], we retained the human capital approach, which is in accordance with the German and international recommendations for performing health economic evaluations [59–61]. The major strength of the study remains its large sample size of 192 individuals and caregivers, which is significant given the rarity of TSC, and the timing of this analysis after mTOR inhibitors were licensed for the treatment of various disease manifestations.

## Conclusions

Expenditures among individuals with TSC are high and are driven by the number and severity of disease manifestations. More effective delivery of existing disease-modifying treatments and the development of new therapies may have the potential to substantially reduce the high clinical and economic burden of TSC for patients and our health system. Productivity losses represent a major source of costs, which should be addressed through improved sociomedical support and therapeutic interventions. Efforts should focus on reducing absenteeism from work and providing integrated, centralized care for individuals with TSC.

## Supplementary Information

**Additional file 1.** Methods of cost and price calculation.

## Data Availability

The datasets analyzed during the current study are available from the corresponding author on reasonable request.
