# Supplementary material for: Direct and indirect costs and cost-driving factors in adults with tuberous sclerosis complex: a multicenter cohort study and a review of the literature
Source: Orphanet J Rare Dis. 2021 Jun 2;16:250. doi: 10.1186/s13023-021-01838-w (PMC8170458; doi:10.1186/s13023-021-01838-w)
Supplement: Supplementary file 1 — Additional file 1. Methods of cost and price calculation. [file 13023_2021_1838_MOESM1_ESM.pdf]

Supplementary material

Method of cost and price calculation:

Our analysis of costs associated with health care resource use followed the guidelines proposed by the German "Working Group Methods in Health Economic Evaluation" (Krauth et al., 2005 [1]). These guidelines specifically cover calculation of direct and indirect medical costs. They include proposed valuation rates for health care services from a societal perspective as an approximations of opportunity costs. We inflated all used costs to 2019 levels using the consumer price index for Germany [2].

1) **Direct costs**

a. Medication costs, medical aids and supplies

We calculated medication costs on the basis of the "Arzneimittel-Verordnungsreport" (Schwabe et al., 2019 [3]) according to the guidelines published by Krauth et al. Specifically, when determining drug consumption by means of a patient questionnaire, the specific medication with dosage is used. If only the preparations (without dosage) are noted, the missing dosage information can be replaced by defined daily dose (DDD) [1]. If only rough information on the type of medical supply is available, an average over similar devices can be used as an approximation. This can be done by determining a list of typical products falling under the same category, or by defining a "representative" product. The guidelines by Krauth et al. contain proposed representative valuation sets for medical aids and these supplies such as wheelchairs, bandages, head protectors. For an example, see the Table below (taken from Table 4 in[1], translated):

**product group according to Hilfsmittelverzeichnis**	**Description**	**€ (Euro)**
5	Bandages	80.88
8	Insoles	13.22
10	Crutches	32.03
14	Inhalation and respiratory aids	121.22
17	Compression stocking	37.63
18	Wheelchairs and other patient transport aids	535.98
21	Measurement devices for vital parameters (e.g. non-invasive blood pressure device)	162.83
23	Ortheses	312.45
24	Prostheses	183.75
26	Sitting aids	376.97
31	Orthopedic shoes	40.11
32	Therapy aids (non-motor)	433.13

b. Out-patient and hospital treatment

We used the valuation sets proposed by Bock et al., 2015 [4] and the physician fee scale ("Einheitlicher Bewertungsmaßstab für ärztliche Leistungen") [5] as the basis for out-patient and hospital treatment. Bock et al. provide valuation sets for in-hospital treatment that are based on a health care resource use questionnaire and are calculated pragmatically based on regularly published sources; valuation sets are regularly updated [4, 5]. If specific departmental cost values were not available, the

average cost values per day were used, see Table below (taken from Table 4 in [4], translated):

	All hospital	General hospital	Intensive care	Psychiatric hospital
Valuation set, full admission, in Euro/day	593,04	575,90	1 337,72	339,71
Valuation set, part-time admission, in Euro/day	385,48	374,33		220,81

2) Care grade allowances

We calculated care grade allowances according as follows:

Care grade	Allowance per month in outpatient setting, care provided by family members (Euro)	Allowance per month in outpatient setting, care provided by external service providers or in inpatient setting (Euro)
1	no allowance	no allowance
2	316	948
3	545	1635
4	728	2184
5	901	2703

3) Out-of-pocket costs

We reported OOP expenses explicitly and added them to the total direct healthcare costs when supply-side utilization estimates were not or when expenditures existed beyond the formal healthcare setting, such as alternative and occupational therapies and equipment costs.

4) Indirect costs

We calculated productivity losses due to TSC (days off, inability to work, reductions in working hours, or early retirement) using the human capital approach for patients of working age (i.e., below the age of 67). The mean annual gross wage of EUR 44,964 in 2019 [6] was used to calculate the productivity costs for each patient. For days taken off work, gross wages were calculated as EUR 215.14 per calendar day, and daily income was multiplied by total days off [7].

1. Krauth C, Hessel F, Hansmeier T, Wasem J, Seitz R, Schweikert B: **[Empirical standard costs for health economic evaluation in Germany -- a proposal by the working group methods in health economic evaluation]**. *Gesundheitswesen* 2005, **67**(10):736-746.
2. DESTATIS Statistisches Bundesamt: **[Consumer Price Index] Verbraucherpreisindex**. In.; 2019.
3. Schwabe U, Paffrath D, Ludwig W-D, Klauber J: **Arzneiverordnungs-Report 2019**: Springer-Verlag Berlin Heidelberg; 2019.
4. Bock JO, Brettschneider C, Seidl H, Bowles D, Holle R, Greiner W, Konig HH: **[Calculation of standardised unit costs from a societal perspective for health economic evaluation]**. *Gesundheitswesen* 2015, **77**(1):53-61.
5. **Online-Version des EBM** [www.kbv.de/html/online-ebm.php]
6. **DESTATIS Statistisches Bundesamt** [www.destatis.de]
7. Strzelczyk A, Nickolay T, Bauer S, Haag A, Knake S, Oertel WH, Reif PS, Rosenow F, Reese JP, Dodel R *et al*: **Evaluation of health-care utilization among adult patients with epilepsy in Germany**. *Epilepsy Behav* 2012, **23**(4):451-457.
